# Fish ecotyping based on machine learning and inferred network analysis of chemical and physical properties

**DOI:** 10.1038/s41598-021-83194-0

**Published:** 2021-02-12

**Authors:** Feifei Wei, Kengo Ito, Kenji Sakata, Taiga Asakura, Yasuhiro Date, Jun Kikuchi

**Affiliations:** 1 RIKEN Center for Sustainable Resource Science, 1-7-22 Suehiro-cho, Tsurumi-ku, Yokohama, 235-0045 Japan; 2 Graduate School of Medical Life Science, Yokohama City University, 1-7-29 Suehirocho, Tsurumi-ku, Yokohama, 230-0045 Japan; 3 Graduate School of Bioagricultural Sciences and School of Agricultural Sciences, Nagoya University, 1 Furo-cho, Chikusa-ku, Nagoya, 464-8601 Japan

**Keywords:** Data mining, Machine learning, Network topology, Metabolomics

## Abstract

Functional diversity rather than species richness is critical for the understanding of ecological patterns and processes. This study aimed to develop novel integrated analytical strategies for the functional characterization of fish diversity based on the quantification, prediction and integration of the chemical and physical features in fish muscles. Machine learning models with an improved random forest algorithm applied on 1867 muscle nuclear magnetic resonance spectra belonging to 249 fish species successfully predicted the mobility patterns of fishes into four categories (migratory, territorial, rockfish, and demersal) with accuracies of 90.3–95.4%. Markov blanket-based feature selection method with an ecological–chemical–physical integrated network based on the Bayesian network inference algorithm highlighted the importance of nitrogen metabolism, which is critical for environmental adaptability of fishes in nutrient-rich environments, in the functional characterization of fish biodiversity. Our study provides valuable information and analytical strategies for fish home-range assessment on the basis of the chemical and physical characterization of fish muscle, which can serve as an ecological indicator for fish ecotyping and human impact monitoring.

## Introduction

**Figure 1:**
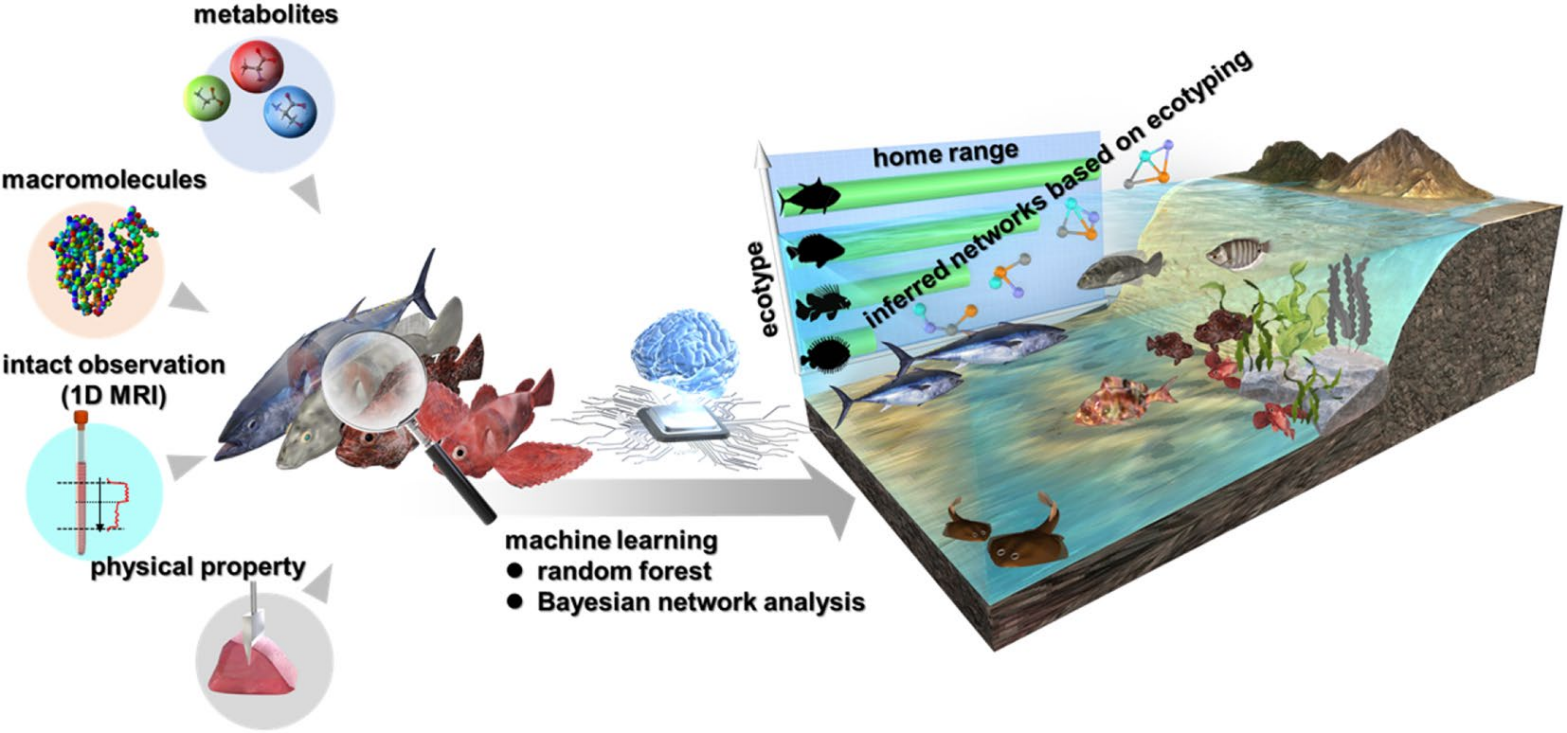
The scheme for the present study. Fish sampling was conducted in Japan at a nationwide scale with Tokyo Bay as the center, covering a wide range of seas and rivers from Hokkaido to Okinawa. A total of 1867 fishes belonging to 249 species were collected from 2011 to 2019. Fish muscle was dissected and prepared for analysis under identical conditions. The functional traits of fish muscle were evaluated with multiple methods, including the biochemical profiling of water-soluble small-molecule metabolites and macromolecules with NMR spectra, physical characteristic assessment with autographs, and intact phenotype observation with 1D MRI. Two data mining strategies were conducted to evaluate fish diversity in this study: (1) machine learning based on the chemical profiles of 1867 fish muscle samples to establish a predictive model for the ecological characterization of the movement patterns and home ranges of fish and (2) the use of BN-algorithm-inferred ecological category-dependent metabolic networks of machine-learned chemical features combined with Markov blanket-based feature selection for an integrated network of chemical (NMR-based small-molecular metabolites and macromolecule composition profiles), physical (stress testing) and phenotypic (1D MRI-based intact observations of texture features) data and ecological categories (migratory, territorial, rockfish, and demersal) to extract the hidden patterns and interactions related to the functional diversity of fish.

With the growing challenges in global food security, it is critical to achieve a sustainable protein supply from both environmental and ecological perspectives. Livestock products are almost completely grown under artificial conditions. However, the feeding and cultivation of livestock might suffer from the inefficient use of energy and water. In contrast, fishes are the most diverse vertebrates with more than 34,000 species^1^. Approximately 30% of the marine biomass is composed of fish species, which provide a critical food source and animal protein to meet the nutritional demands of billions of people^2^. For example, there is growing interest in *sashimi* and *sushi* products, which are traditional Japanese seafoods consisting of fresh raw fish, as they are low-calorie and healthy foods. The biodiversity of fish has been shown to be an important factor in promoting the production of fish biomass^3^. In the marine environment, fishes quickly respond to multiple environmental stresses, such as climate change and pollution, causing disturbances in species composition, diversity, and ecosystem function^4^. There is growing recognition that the quantification of the functional and phenotypic variations among species, such as the measures of functional diversity, rather than species richness, is critical for measuring species diversity and understanding ecological patterns and processes^5^. Functional traits are defined as any measurable features of an individual who could affect the persistence and performance of a species and its ecological interactions within a community, which could be physical, biochemical, behavioral, or phenological, among other effects^6^. Recent studies have provided evidence that in comparison with taxonomic diversity, the functional diversity of fish could be more sensitive to environmental stresses and might serve as a better predictor of ecosystem function^7^. However, given the large number of species and the complicated species interactions and ecosystem dynamics, it is still challenging to predict and quantitatively characterize the functional diversity of fish at a large scale^8^. A metabolome is a collection of the final product of all cellular activities, and it reflects the complex interactions among endogenous biological processes and environmental stimuli. The changes in metabolites are often more sensitive than those in RNA transcripts or proteins to biological and environmental changes. This difference makes metabolomics a relevant tool for environmental monitoring and safety assessments^9^. The nondestructive profiling of the chemical matrix using nuclear magnetic resonance (NMR) spectroscopy, which does not require chemical separation, provides an ideal approach to explore the complicated interactions of genes, growth stages and the surrounding environment of wild fish in their natural state^10–13^. Advances in data science and big data technologies, such as machine learning, deep learning and artificial intelligence, have enabled the extraction of useful information by evaluating the relative importance of each feature according to model coefficients and the establishment of simulation models to predict ecosystem dynamics on the basis of high dimensionality and large data sets in which raw data are largely unlabeled and uncategorized^12,14–16^. Successful applications of big data analysis have mainly involved nonbiological data, such as in the fields of atmospheric physical movement (weather forecasting) and market analysis. Notably, studies have been limited by ease of access to massive amounts of data; for example, electronic sensors can be easily used to record physical environmental information such as the temperature, humidity, wind velocity and direction, and precipitation, and trade details and market transaction information can be obtained from the internet^17^. However, applications of big data analysis in computational ecological modeling are still limited. The main bottleneck is the difficulty related to large-scale biological sampling and analysis under identical conditions. Indeed, it is well known that machine learning approaches are quite beneficial for learning from large volumes of unsupervised data. Biochemical and physical processes consist of systems of interacting molecules and macromolecules. One excellent approach that can be used to integrate individual features into a meaningful network that might represent physical interactions and remove transitive relationships is to infer a network by modeling the dependencies among variables^18^. There is growing interest in the application of the Bayesian network (BN) inference algorithm in environmental modeling and management, such as in predicting population dynamics for a single species and assessing the functional relationships between species and habitats within ecosystems^19,20^. The BN inference algorithm, which represents the joint posterior probability distribution over the whole set of variables in a system, is considered more biologically interpretable than correlation networks that consider each relationship independently^21^. In comparing algorithm results with observational data from traditional field studies, the BN inference algorithm has proven to be a powerful tool in ecosystem analysis that can accurately reveal known relationships and identify key features with high connectivity within an ecosystem by inferring the network structure^22^. Therefore, a BN-inferred functional network architecture provides a valuable solution to process and interpret the outcomes of machine learning models from biological perspectives^23^. Collectively, the main motivation for the present study is the lack of a perspective on the functional diversity of fish and the corresponding relationship with the ecological characteristics of the natural state over a large scale. Therefore, this study aimed to develop novel integrated analytical strategies for the functional characterization of fish diversity based on the quantification, prediction and integration of the chemical and physical variations in fish muscles (Fig. 1). Fish sampling was conducted in Japan at a nationwide scale, large-scale biochemical fish muscle data were generated using NMR, and physical fish muscle data were generated by examining muscle strength using stress testing, an autograph and observations of intact textural features with one-dimensional magnetic resonance imaging (1D MRI). Machine learning models run with the improved random forest (RF) algorithm based on 1867 muscle NMR spectra for 249 fish species successfully predicted the mobility patterns of fishes into four categories (migratory, territorial, rockfish, and demersal) with accuracies of 90.3–95.4%. Eleven important muscle metabolites, including creatine, histidine, lactate, inosine, glutamine, *N*-acetyl-glutamate (NAG), carnitine, taurine, alanine, proline, and uridine diphosphate (UDP)-glucose, were extracted according to the Gini index. Then, ecological category-dependent metabolic networks of the machine-learned chemical features and Markov blanket-based feature selection for an ecological–chemical–physical integrated network were established with the BN inference algorithm to mine the functional connections among the high-dimensional data factors. Collectively, our study provides valuable information and analytical strategies for fish home-range assessment on the basis of the chemical and physical characterization of fish muscle, which can serve as an ecological indicator for fish ecotyping and human impact monitoring.

## Results and discussion

### Fish sampling and data generation

Fish sampling centered in Tokyo Bay was conducted from 2011 to 2019, and it covered a wide range of seas and rivers from Hokkaido to Okinawa. A total of 1867 fishes belonging to 249 species were collected with no restrictions on fish species or environmental conditions (Table S1). Fish muscle was dissected and prepared for analysis under identical conditions. Fish muscle was the focus of this study because it is the main edible part of fish and reflects the athletic ability of fish, which is closely related to the ecological characteristics of their habitat and life habits^14^; additionally, muscles are the main protein source of fish. Large-scale data sets of the chemical composition, physical characteristics and phenotypical microstructure of fish muscle were generated by NMR spectroscopy, autography and 1D MRI, respectively. The impacts of diverse factors in the system were examined at the level of the data structure to filter out noise and mine meaningful information/patterns of interest. This data-driven model was constructed by filtering the noise from the data structure without the need for additional measurements. Therefore, the technical demands on the experimental system were not considerable, making it possible to apply the method under nonhypothetical conditions to a wide range of objects/systems, including ecosystems. Two data mining strategies were implemented to evaluate fish diversity in this study: (1) machine learning based on the chemical profiles of 1867 fish muscle samples to establish a predictive model for the ecological characterization of movement patterns and habitat use of fish and (2) the use of BN-algorithm-inferred ecological category-dependent metabolic networks of machine-learned chemical features combined with Markov blanket-based feature selection for an integrated network of chemical (NMR-based small-molecular metabolites and macromolecule composition profiles), physical (stress testing) and phenotypic (1D MRI-based intact observations of texture features) data and ecological categories (migratory, territorial, rockfish, and demersal) to extract the hidden patterns and interactions related to the functional diversity of fish.

### Machine learning-based ecological prediction using large-scale metabolomic data

**Figure 2:**
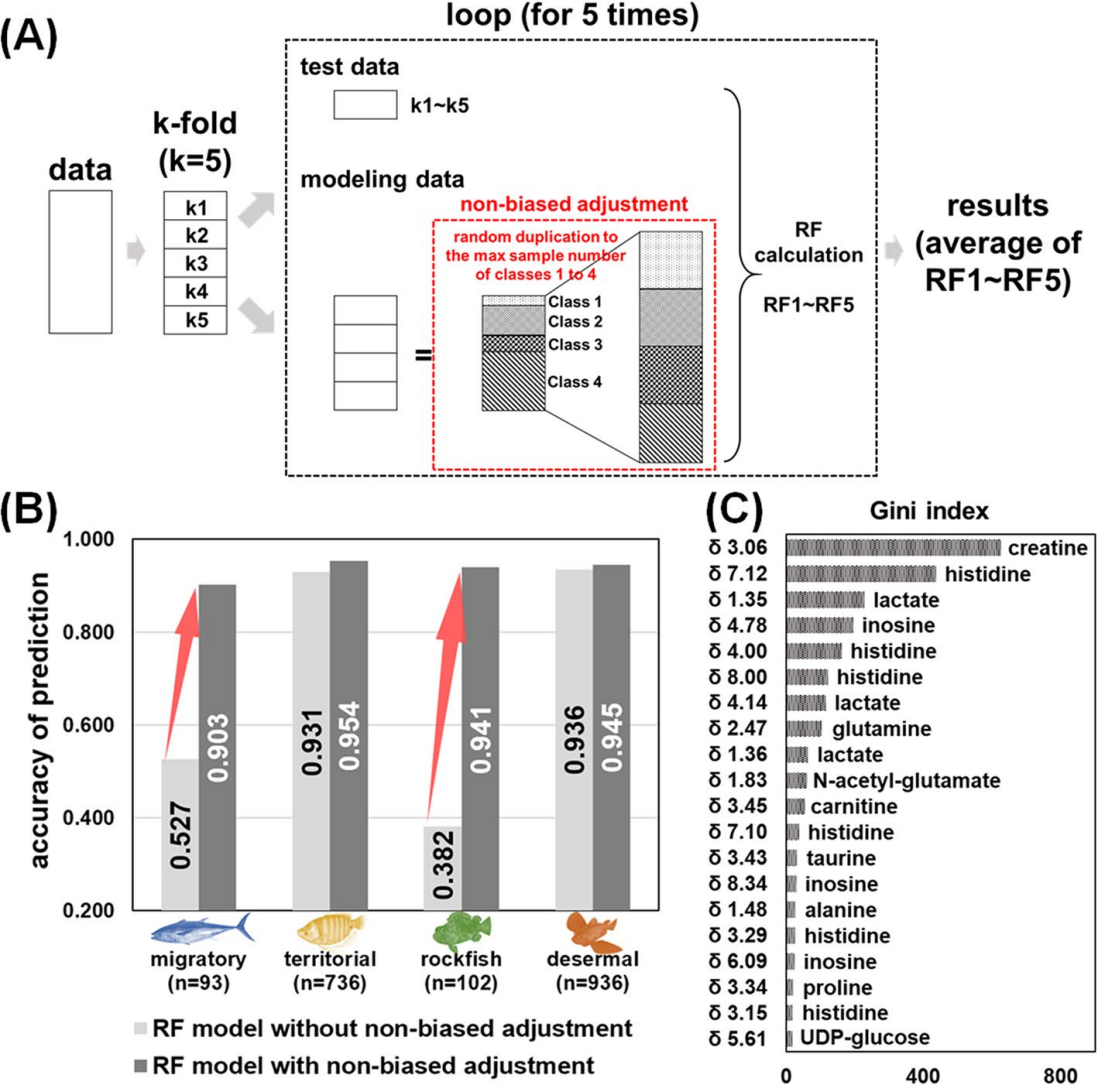
Machine learning for ecological prediction according to the NMR-based chemical profiling of fish muscle. (**A**) Conceptual diagram of the improved random forest (RF) algorithm used in the present study. After the division and reservation of the test data, the data for each class used for modeling were randomly duplicated to reach the maximum sample number for classes 1 to 4 to eliminate bias. The generated data with equal sample numbers for each class were used as the modeling data. RF calculations were performed 5 times each with the test data for k1 to k5. The result files of RF (prediction accuracy and important variables identified by the Gini index) were generated, and the average values of RF1 to RF5 were calculated and used as the final RF results. (**B**) Accuracy of the prediction of fish ecotype using NMR-based machine learning. (**C**) The most important metabolites in the discrimination of fish ecotypes ranked by the Gini index were *δ* and the ^1^H chemical shift.

Unsupervised principal component analysis (PCA) revealed that the most important factor that affects the metabolic profile of fish was environmental salinity, followed by the growth stage and habitat depth, as shown in Fig. S1. The fish mobility pattern and habitat use are critical functional traits that are characterized by ecological features^24^. The fishes used in this study were divided into four categories according to their mobility pattern: migratory, territorial, rockfish, and demersal (Table S1). Muscles are the peripheral structures of the motor system and are used in exercise. The chemical composition and structural characteristics of muscles represent the result of ecological adaptation. Next, based on the water-soluble metabolic profiles of all 1867 fish muscle samples, a large-scale machine learning model was established using RF methodology; this method is an ensemble learning algorithm used for classification, regression, and clustering^25^ and was applied for the prediction of the mobility patterns of fish ecological characteristics. Due to the unbalanced distribution of fishes in the four ecological categories (migratory: 93 fishes; territorial, 736 fishes; rockfish: 102 fishes; and demersal: 936 fishes), RF predictive modeling for categories with a small number of samples might lead to inadequate learning, subsequently resulting in the prediction accuracy being largely dependent on the distribution of learning samples (Fig. S2). To address this issue, we improved the RF algorithm following the scheme shown in Fig. 2A. In detail, the data were divided into two modules: the test module and learning module. After assessing the prediction accuracy in the test module, the bias of the sample was adjusted by randomly duplicating the data in the learning module. Notably, the improved RF model effectively enhanced the accuracy of the prediction of the ecological characteristics of fish to as high as 90% in all four categories based on the water-soluble metabolic profiles of fish muscle (Fig. 2B). Accordingly, the impact of each NMR peak on the predictive power of the RF model was evaluated based on the Gini index ranking (Fig. 2C). Peak assignment revealed that fish muscle metabolites such as creatine, histidine, lactate, inosine, glutamine, and NAG had high Gini scores, suggesting that these metabolites are important in the prediction of the mobility patterns of fish. For example, high levels of metabolites involved in energy metabolism, such as creatine, lactate, and UDP-glucose, were observed in the muscles of oceanodromous fish species (Fig. S3). A relatively high distribution of histidine, which is an intracellular proton buffering constituent required for anaerobic performance, such as burst swimming in fishes^26^, was observed in the muscles of migratory fishes (Fig. S3).

### Ecological category-dependent network of machine-learned chemical features using the BN inference algorithm

**Figure 3:**
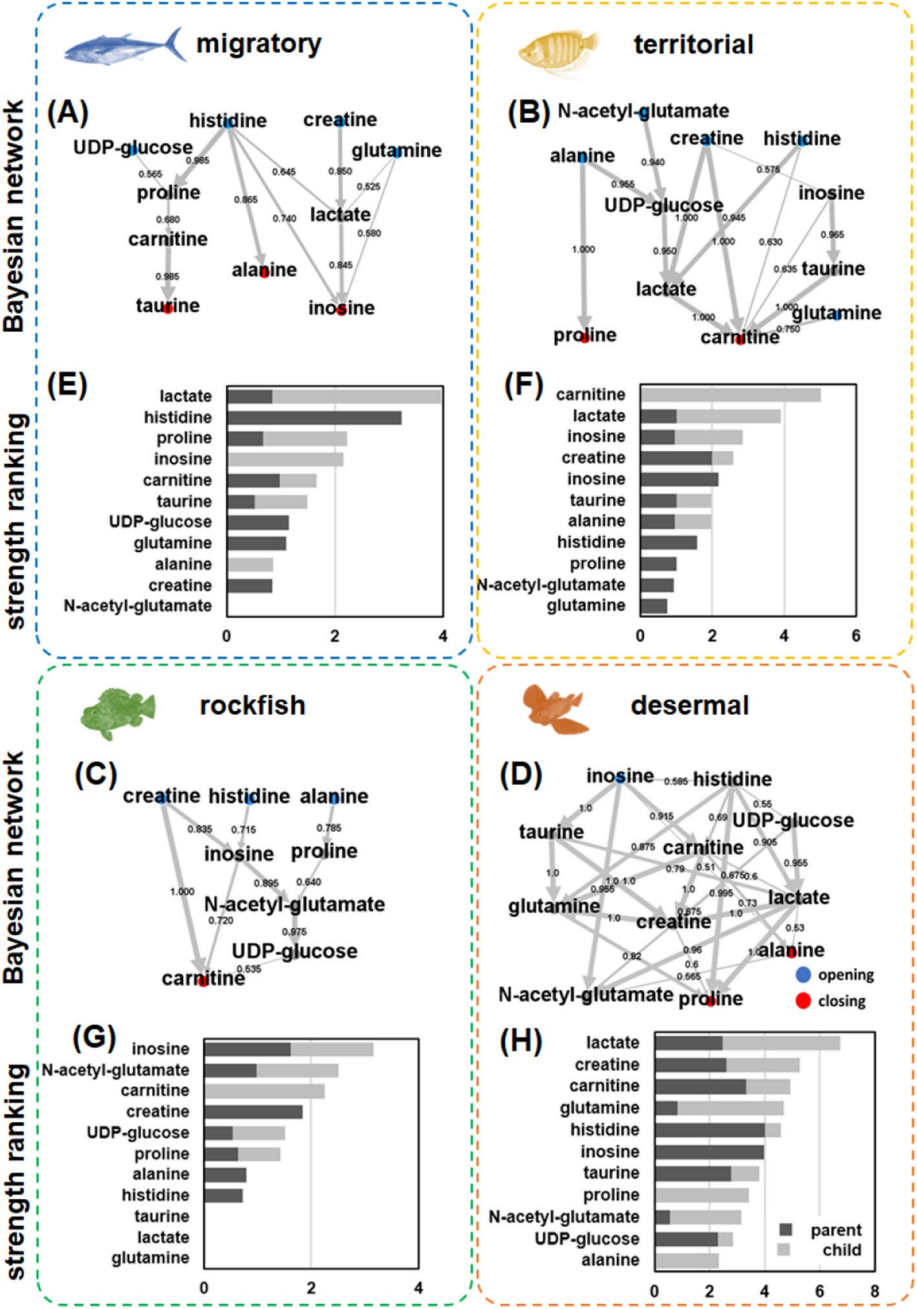
Ecological category-dependent (**A**)–(**D**) directed acyclic graphs (DAGs) and (**E**)–(**H**) the rank of variable importance using the BN inference algorithm. DAGs were reconstructed with the machine-learned chemical features of migratory, territorial, rockfish and demersal fish, respectively. (**A**)–(**D**): the circular network diagrams display all the network edges with a strength of probabilistic dependence greater than 0.5 between any of the muscle metabolites. Red: beginning nodes without parent nodes; blue: end nodes without any child nodes. (**E**)–(**H**): the importance of each variable was calculated as the sum of all the probabilistic strengths of each variable connected to the corresponding parent (in black) and child (in gray) nodes.

To recover meaningful networks of functional relationships from machine-learned metabolic data, BN analysis was performed on the important muscle metabolites (creatine, histidine, lactate, inosine, glutamine, NAG, carnitine, taurine, alanine, proline, and UDP-glucose) with high Gini scores in each ecological category using the hill-climbing algorithm in the bnlearn R package. The reconstructed BN structures involved circular network diagrams that displayed all network edges with a conditional dependence level greater than 0.5 between any of the muscle metabolites (Fig. 3A–D). Topological analysis demonstrated structural diversity among the four ecological categories. Notably, the creatine node with the highest Gini score in the RF model was located at the top of the network hierarchy for migratory, territorial, and rockfish fishes but was located in the center of the network for demersal fish (Fig. 3A–D). Creatine is a key product of the energy compound creatine phosphate in the so-called Lohmann reaction and is an indicator of muscle performance^27^. Creatine-related network topological diversity might reflect the functional differences in movement patterns between demersal fishes and other fishes. Next, the importance of each variable was calculated as the sum of all the probabilistic strengths of the variables connected to the corresponding parent (red) and child (blue) nodes (Fig. 3E–H). A comparison of importance ranks using Kendall’s coefficient of concordance showed large diversity across the four ecological categories (Kendall’s *W* = 0.34, *P* = 0.19). Of interest, low rankings for glutamine were observed in migratory, territorial, and rockfish species, while a relatively high rank for glutamine was observed in demersal fish. To further investigate the biological basis of fish biodiversity, metabolic pathway analysis was performed on the top-6-ranked metabolites of each ecological category using the online platform Metaboloanalyst^28^. Pathway enrichment analysis using Metaboloanalyst showed that “ammonia recycling” was the most significantly enriched metabolite set in the muscle of demersal fish but not that of the other three fish types (Fig. S4). These data highlighted the importance of nitrogen metabolism in the functional characterization of fish biodiversity. Notably, the ammonia detoxification ability of fish is critical for environmental adaptability in ammonia-rich environments that are affected by various industries and human activities^29^. Coastal hypoxia, which is induced by an increase in nutrient inputs attributable to anthropogenic origins, fundamentally alters the diversity and functionality of coastal fishes across the land–sea interface^30^. It is reasonable to consider that variations in the functional trials of fish species, especially demersal fish species in nutrient-rich environments attributable to human activities, are largely dependent on the level of human impact. The above data suggested that the BN structures exhibited preserved patterns and complicated relationships among metabolites belonging to the same ecological category, which inspired us to examine whether the dependence among metabolites might be related to fish biodiversity. To explore this hypothesis, 200 fish individuals were randomly selected 200 times to generate 200 metabolic datasets for BN analysis. For each dataset, the strength of the probabilistic dependence between each of the 11 important muscle metabolites and the Shannon diversity index across the four ecological categories (migratory, territorial, rockfish, and demersal) were calculated. To achieve the most discrete distribution of fish diversity, the 10 datasets with the largest and smallest Shannon index values were selected. Then, the metabolic dependence related to fish biodiversity was selected according to the Pearson correlation between the strength of the conditional dependence of each BN edge and the Shannon index. Hierarchical clustering demonstrated the strength of the highly correlated metabolic dependence (Fig. S5). Notably, the strength of the conditional dependence between histidine and lactate was retained across the diverse datasets. The serine–histidine–aspartate motif is one of the most thoroughly characterized catalytic motifs in biochemistry. The aspartate hydrogen bonded to histidine, which increases the p*K*_a_ of imidazole nitrogen from 7 to approximately 12, results in histidine acting as a powerful general base and activator of other nucleophiles, such as serine or cysteine^31^. In addition, the strength of conditional dependence associated with ornithine-urea cycle (OUC) metabolites, such as NAG and glutamine, increased with the Shannon index. Since the joint probability of BNs indicates the likelihood of two events occurring together and equivalently disappearing together, these data suggest that nitrogen metabolism-related dependence might be a determinant factor in the functional characterization of fish diversity.

### Multidimensional profiling of fish muscles in species with different ecological characteristics

**Figure 4:**
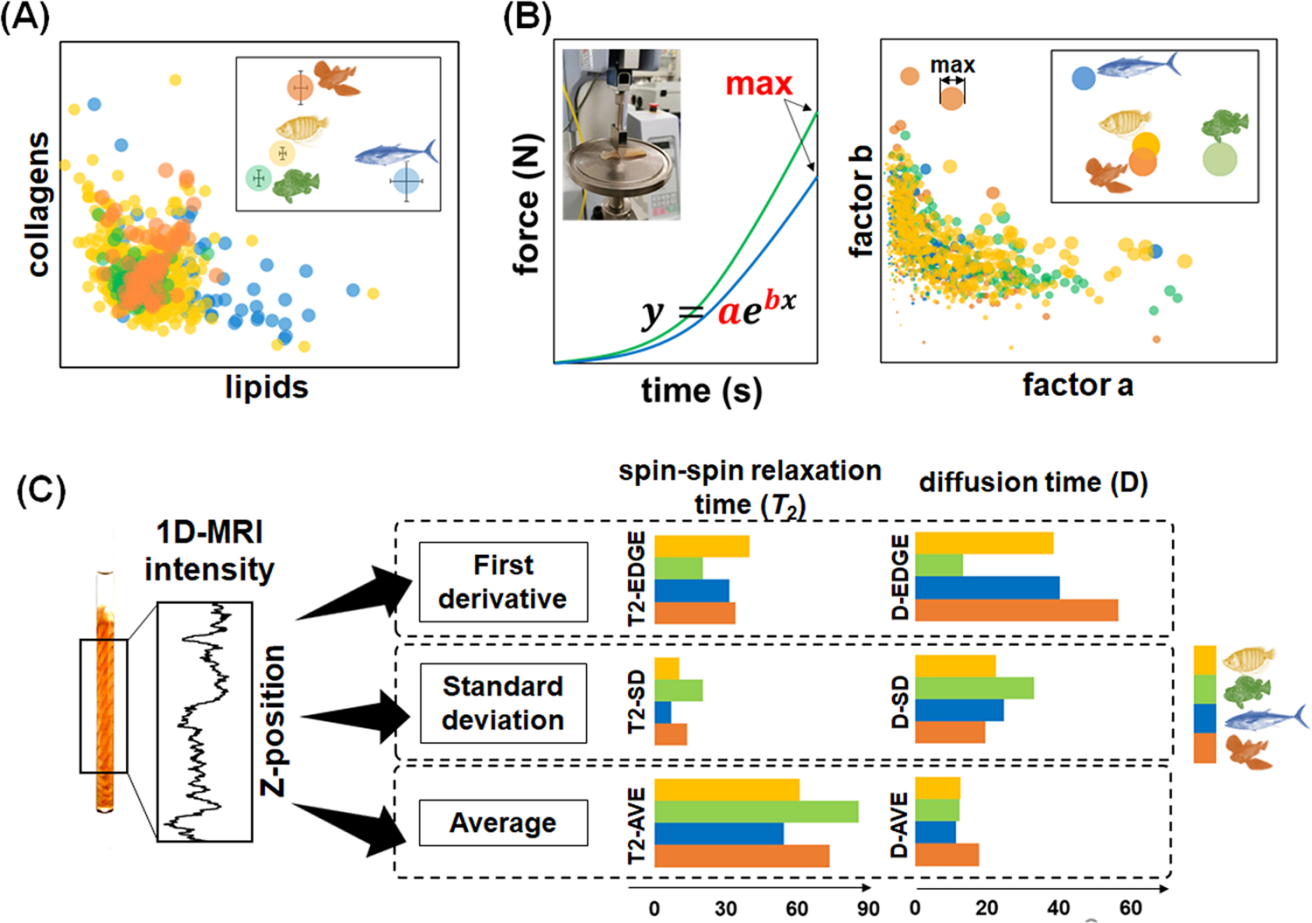
Multidimensional profiling of fish muscles in species with different ecological characteristics. Distribution of (**A**) the major macromolecules, (**B**) index of the exponential function of the fitted cut-off stress curve, and (**C**) 1D MRI features of fish muscle samples among the four ecological categories of fish: migratory, territorial, rockfish, and demersal. The insert in (**A**) indicates the average value of each ecological category. The error bar presents standard error. The left panel in (**B**) shows a representative photo and the force–time curve of fish muscle stress testing using an autograph. The three exponential function parameters, the *y*-intercept parameter *a* and the appropriateness factor *b* of the natural exponential base, and the maximum force value (*max*) of force–distance curve are highlighted in red. The right panel in (**B**) shows the distribution of the parameters *a* and *b* of each ecological category, which are scaled based on the parameter *max* values. The insert of the right panel in (**B**) shows the average value of each ecological category. The left panel in (**C**) shows the drilled cylindrical muscle was inserted into a 5-mm NMR tube filled with KPi buffer. The observation area of the *Z*-axis gradient was approximately 150 mm of the equipped probe used in the present study. 1D MRI signals of proton density, *T*_2_ and diffusion coefficients were observed. After calculating the moving averages of ± 100 data points, the first derivative of the curve was calculated to evaluate the “EDGE” (number of zero points in the first derivative) of the 1D imaging data. The right panel in (**C**) shows the distribution of 1D MRI features of fish muscle samples of each ecological category.

Then, the major soluble macromolecule composition, physical characteristics and phenotypic textural features of fish muscles were examined, and the corresponding distributions were evaluated among different ecological categories. We extracted the signal profiles of the major water-soluble macromolecular components from the performance baselines using an integrated analytical strategy that combined covariation peak separation and matrix decomposition and identified two major macromolecules, lipids and collagens, in the fish muscle extracts^32^. Here, we examined the distribution of these macromolecules among the four ecological categories (Fig. 4A). Relatively high distributions of lipids and collagens were observed in the muscles of migratory fish and demersal fish, respectively. Low contents of both lipids and collagens were observed in the muscles of rockfish. Next, to gain insight into the physical properties of fish muscle, the compressive force was examined, and exponential fitting was performed on the force–stroke curves (Fig. 4B). Three exponential function parameters, the *y*-intercept parameter *a* and the appropriateness factor *b* of the natural exponential base, and the maximum force value (*max*) of force–distance curve were used to describe the trends of the force–stroke curves and enable an accurate characterization of the physical properties of muscle tissues in response to external cutting forces. The plot of the fitting coefficients for *a* and *b* indicated a strong correlation between the physical properties of fish muscle and the mobility patterns. The muscles of migratory fish were characterized by an exponential curve with a relatively small *a* value and a relatively large *b* value (the stress curve grew at a late point and then quickly increased), suggesting easy compression but lack of complete cutting. In contrast, the muscles of rockfish were characterized by an exponential curve with a relatively large *a* value and a relatively small *b* value (the stress curve quickly increased in the early stage and then slowly increased), suggesting that the muscle was easily cut but not easily compressed. Therefore, parameters *a* and *b* could reflect the physical characteristics of fish meat in terms of elasticity (a rubbery mouthfeel) and softness (a residual chewy mouthfeel). Then, intact phenotypic observations were performed on fish muscle using 1D MRI^33^. Unlike the averaged signals in the conventional NMR spectra, changes in the tissue characteristics could be detected in 1D MRI based on the spatial position of the *Z*-axis (Figs. 4C, S6). We performed statistical processing for the average (AVE), standard deviation (SD), and first derivative (number of edges; EDGE) of the variation curves of the transverse relaxation time *T*_2_ and diffusion coefficients (*D*), which depended on the spatial position of the *Z*-axis (Fig. 4C). As a result, demersal fish and rockfish muscle had relatively large AVE and SD values for *T*_2_. Rockfish muscle had relatively small EDGE values for *T*_2_. Demersal fish muscle had relatively high *D* and *D* EDGE values, and rockfish muscle had the smallest *D* EDGE value. Collectively, the biochemical, physiological and structural properties of fish muscles were highly correlated with ecological characteristics, such as the movement patterns and habitat use of fish.

### Markov blanket-based feature selection from a BN-inferred ecological–chemical–physical integrated network

**Figure 5:**
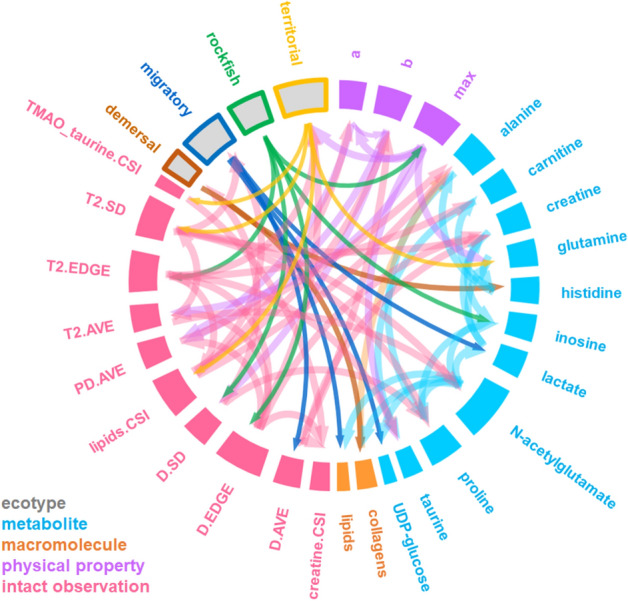
Integrative network of chemical, physical and phenotypic profiles of fish muscle and ecological categories according to the BN inference algorithm. NMR-based low-molecular-weight chemical data were used to establish continuous variables. The discretized data for the major soluble macromolecule composition, physical characteristics and 1D MRI-based phenotypic textural features for fish in the four ecological categories (migratory, territorial, rockfish, and demersal) were used as categorical variables. The integrated network was generated using the score-based hill-climbing learning algorithm in the bnlearn R package. The length of node bars was scaled according to the number of connected edges. The nodes were colored according to the measurement techniques and ecological categories. “PD”: 1D MRI factor for the proton density; “*T*_2_”: spin–spin relaxation time; “D”: diffusion coefficient; “SD”: standard deviation; “EDGE”: number of zero points in the first derivative of 1D MRI data; and “CSI”: chemical shift imaging, as described in Fig. S6.

**Figure 6:**
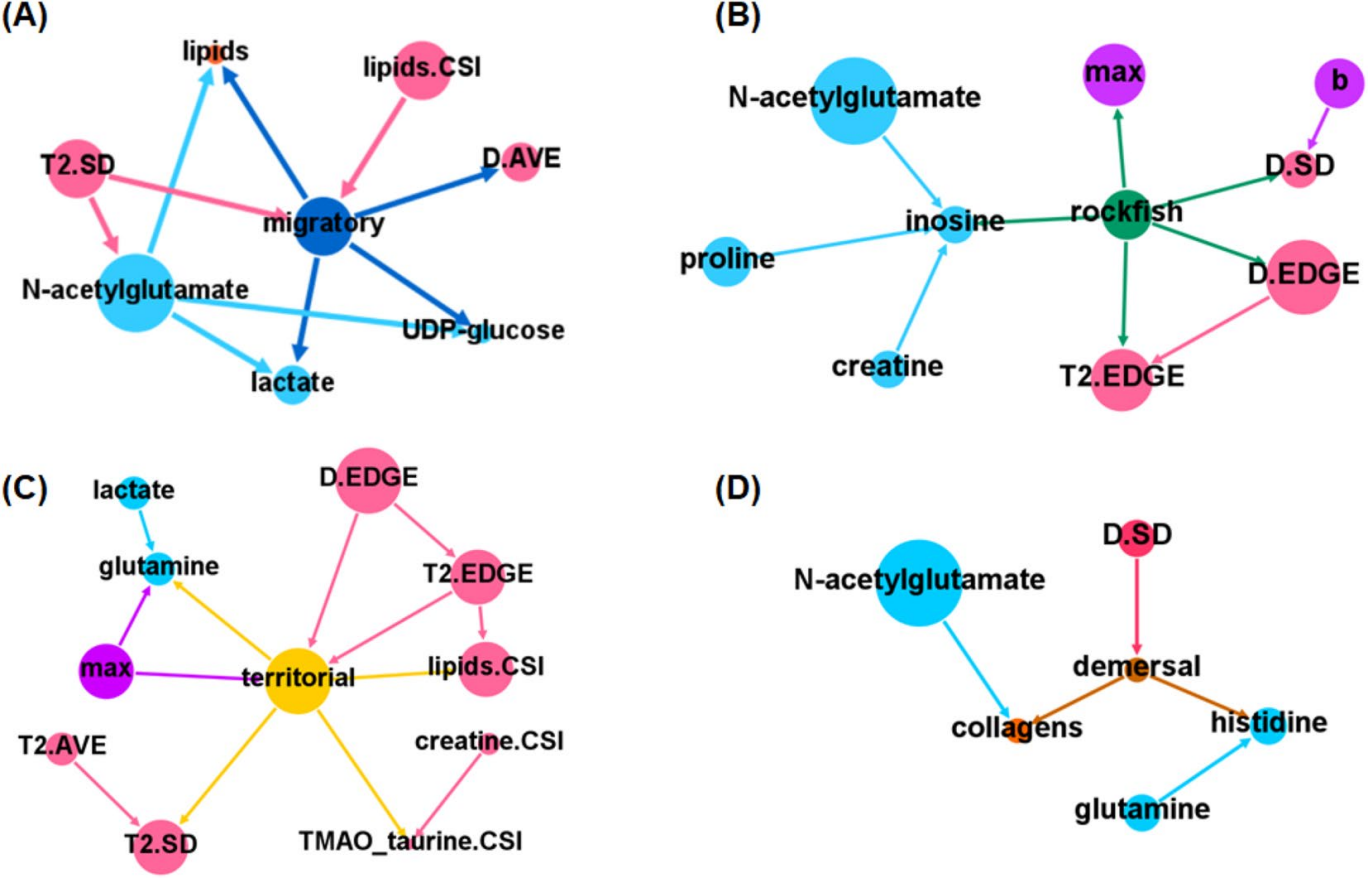
Markov blanket-based feature selection from the BN-inferred ecological–chemical–physical integrated network. Markov blankets of the nodes (**A**) migratory, (**B**) territorial, (**C**) rockfish and (**D**) demersal fish represent the union of the parents, children, and other parents of those children. The arrows are colored by parents. The node size is based on edge weights according to the integrative network of Fig. 5.

As a probabilistic modeling approach, the BN inference algorithm enables the integration of complex heterogeneous data, including categorical and continuous data, from different sources to assess the relationships between multiple environmental factors and ecological indicators^34^. Therefore, all the data obtained from multiple techniques and ecological categories were integrated using the BN inference algorithm to extract the hidden patterns and interactions related to the functional diversity of fish. The generated ecological–chemical–physical integrated network with a score-based hill-climbing algorithm identified multiple highly correlated features that might play dominant roles in shaping fish diversity (Fig. 5). Overall, the integrated network demonstrated that NAGs were at the central location in the network and had the largest number of connections. An integrated network analysis of multidimensional data demonstrated the critical role of nitrogen metabolism in the functional characterization of fish biodiversity. In addition, the integrated network provided other valuable clues for the functional understanding of fish diversity. Taurine is known to play a critical nutritional role in the growth and development of marine fish. Dietary taurine supplementation might increase the growth rates of fish and decrease motility due to its role in hemolytic suppression through osmoregulation and biomembrane stabilization in fish^35^. The taurine contents in muscle were correlated with the maximum value of the force–distance curve, suggesting that the distribution of taurine in natural fishes is closely related to muscle strength. It is possible that fish species with high muscle strength would require more muscle taurine than other species to maintain the stabilization of muscle cell membranes. According to the force–distance curve of fish muscle, the parameter *a* value will increase at an early stage, reflecting a rubbery mouthfeel. In contrast, the parameter *b* value will increase in a later stage, reflecting a residual chewy mouthfeel. Our integrated network analysis showed that parameter *b* was correlated with parameter *a* and the SD of the position-dependent diffusion curve (*D.* SD) obtained from the 1D MRI of fish muscle. This result suggests that the degree of fluctuation of the apparent diffusion coefficient (ADC), which measures the rate of diffusion of water molecules within a tissue^36^, is related to parameter *b*. The ADC in biological tissues is determined by multiple factors, such as the cell type and density. Therefore, the fluctuation in the ADC, which is presented as the SD value of ADC, could reflect the degree of heterogeneity in the samples. Collectively, the force–distance curve of fish muscle showed that the rubbery mouthfeel of fish muscle is probably related to the strength of the muscle cell membrane, while the residual chewy mouthfeel of fish muscle might be related to the connective tissue interspersed between myocytes. Finally, to further understand and characterize the biodiversity of fish at multiple levels, Markov blanket-based feature selection was applied at the node level in each ecological category (Fig. 6). A Markov blanket was defined as the union of the parents (nodes connected above), children (nodes connected below), and other parents of those children for identifying redundant and irrelevant features of the node of interest in the reconstructed BN^37^. Remarkably, the Markov blanket of migratory fish was connected to metabolites involved in energy metabolism, such as lactate, UDP-glucose and muscle lipids, representing advanced swimming ability during migratory movement. In addition, the Markov blanket of dermal fish was connected to the OUC metabolites NAG and glutamine, which was in accordance with our above observations that demersal fish were characterized by their ammonia detoxification ability in nutrient-rich environments. These data suggest that the Markov blanket-based feature selection of BN structures provides a powerful approach for extracting fundamental knowledge about the functional characterization of fish biodiversity from multidimensional data.

In summary, the biochemical, physical and phenotypic profiles of fish muscles from 1867 individuals of 235 species were comprehensively evaluated using multiple techniques, including NMR, autograph testing and 1D MRI. Consequently, the diversity of fishes in terms of their mobility pattern and home range was predictively and functionally characterized based on big data collection with PCA, machine learning, and BN-inferred network analysis methods. The NMR-based chemical profiling of fish muscles was performed to classify the fishes according to their habitat features, such as salinity and depth. Machine learning models with nonbiased adjusted RF algorithms successfully predicted the mobility patterns of fishes into four categories (migratory, territorial, rockfish, and demersal) with accuracies of 90.3–95.4% based on the metabolic profiles of fish muscles. Muscle metabolites such as creatine, histidine, lactate, inosine, glutamine, NAG, carnitine, taurine, alanine, proline, and UDP-glucose were identified as the most important factors according to the Gini index for the prediction of fish mobility patterns. Then, the functional features of each ecological category were extracted from the BN-inferred ecological category-dependent metabolic network of the machine-learned chemical features and the ecological–chemical–physical integrated network for Markov blanket-based feature selection. Notably, our findings distinguished demersal fish from those in other ecological categories, highlighting the critical roles of nitrogen metabolism and ammonia detoxification in the functional characterization of fish biodiversity. In addition, ammonia detoxification is closely related to the environmental adaptability of fishes in nutrient-rich environments attributable to human activities, which can be used for assessing the consequences of global changes resulting from human activities and maintaining seafood sustainability for humans. Collectively, our study provides valuable information and analytical strategies for fish home-range assessment on the basis of the chemical and physical characterization of fish muscle, which can serve as an ecological indicator for fish ecotyping and human impact monitoring.

## Materials and methods

### Sample collection and preprocessing

Natural fish samples (n = 1867 individuals belonging to 2 classes, 23 orders, 82 families, 171 genera, and 249 species) were collected over a period of nine years from May 2011 to August 2019 from 33 inland, estuarine and coastal regions of the water ecosystem in Japan, as shown in Table S1. No specific permission was required at any of the sampling points, as they were all in public areas. The animal experiments were performed in accordance with protocols approved by the Institutional Committee of Animal Experiments of RIKEN and adhered to the guidelines of the Institutional Regulation for Animal Experiments and Fundamental Guidelines for the Proper Conduct of Animal Experiments and Related Activities in Academic Research Institutions under the jurisdiction of the Ministry of Education, Culture, Sports, Science and Technology, Japan. This study was carried out in compliance with the ARRIVE guidelines (http://www.nc3rs.org.uk/page.asp?id=1357). After dissection, the fish muscle was collected and prepared for different analyses. For compressive force measurements, fish muscle above the anal fin was picked and cut into slices (approximately 5 mm thick and 10 mm wide, as shown in Fig. 3B). For intact observations by 1D MRI, a stainless pipe (*Φ*_*inside*_ = 4 mm, as shown in Fig. S6) was used as a cutter. The drilled cylindrical muscle was inserted into a 5-mm NMR tube filled with 100 μl of KPi (0.1 M K_2_HPO_4_/KH_2_PO_4_ in D2O, pH 7.0) with 1 mM of sodium 2,2-dimethyl-2-silapentane-5-sulfonate (DSS) as an internal standard and centrifuged at 3000 rpm for 10 min for degasification. For NMR observations of metabolites and major macromolecules, the remaining fish muscle was lyophilized and crushed into powder. Eighteen milligrams of each powdered sample was extracted with 600 μl of KPi at 65℃ for 15 min and centrifuged at 14,000 rpm for 5 min. The supernatant with 1 mM of DSS was transferred to a 5-mm NMR tube.

### Physical property analysis

Stress testing was performed using a multipurpose stretching tester that included an autograph (EZ-L, Shimadzu Co. Ltd., Kyoto, Japan) with a wedge-shaped cutter bit, as shown in Fig. 3B. The loading rate was 2 mm min^−1^, the total distance was 5 mm, and the force–time curves were recorded. The force–time curves for fish muscle were exponentially fit in Microsoft Excel using the following equation:

1 $$y=a{e}^{bx},$$

where *x* represents the time from the starting point, *y* represents the compressive force, and *a* and *b* represent the fitting coefficients.

### NMR-based metabolomics

For metabolome observations, two-dimensional *J*-resolved (2D*J*) spectra (pulse sequence of jresgpprqf) were acquired at 298 K using a Bruker AVANCE II 700 spectrometer equipped with a ^1^H inverse triple-resonance cryogenically cooled probe with *Z*-axis gradients (Bruker BioSpin GmbH, Rheinstetten, Germany). The parameters were as follows: data points, 16 K (F2) and 16 (F1); number of scans, 32; spectral widths, 12,500 Hz (F2) and 50 Hz (F1); and acquisition time, 0.66 s (F2) and 0.32 s (F1). The 1D projection of F2 was obtained with Topspin 4.0.6 (Bruker BioSpin GmbH, Rheinstetten, Germany). All baseline 1D projections were collected, and the peaks were identified by rNMR^38^ on the R platform (v. 3.4.4). The peak intensity matrix was normalized by probabilistic quotient normalization (PQN)^39^, scaled, centered using R and then sorted as a basic data matrix.

### NMR observations of major soluble macromolecules

For water-soluble macromolecule observations, a diffusion-edited pulse sequence (ledbpgp2s1d) was used, and the parameters were as follows: gradient strength, 36.6% of the maximum gradient strength (48.15 G/cm); little delta (*δ*), 1.5 ms; big delta (*Δ*), 120 ms; gradient recovery delay, 200 μs; data points, 16 K; number of scans, 128; spectral width, 11,160.71 Hz; and acquisition time, 0.73 s. Information on major soluble macromolecules was extracted based on peak separation^14^ and Moore–Penrose pseudoinversion^32^.

### Intact observation based on 1D MRI

All MRI experiments were performed at 298 K using a Bruker Avance III HD-500 spectrometer equipped with a ^1^H inverse probe with triple resonance (Bruker BioSpin GmbH, Rheinstetten, Germany). The imaging area (range of the *Z*-gradient region) was detected as approximately 150 mm using a series of layered solutions, as shown in Figs. 3C and S6C. As shown in Fig. S7, the pulse sequences for imaging were modified by embedding the *Z*-gradient into standard sequences of the acquisitions^33^. Proton density profiles were obtained with the spin echo imaging scheme depicted in Fig. S7A. The strength of the magnetic gradients was set at 19.26 G/cm (40% of the maximum 48.15 G/cm); spectral width, 250,000 Hz; and acquisition time, 0.001948 s. The approach used to measure the spin–spin relaxation time (*T*_2_) and the diffusion coefficient (*D*) of the fish muscle along the depth-concentration profile is schematically represented in Fig. S6B,C. The parameters were as follows: spectral width of 250,000 Hz and acquisition time of 0.001948 s. To analyze such a series of data, Dynamic Center (Ver 2.5.4, Bruker BioSpin GmbH, Rheinstetten, Germany) was used to calculate *T*_2_ and *D* in the depth profiles. All data points in the imaging area (approximately 4000 data points) were used, and *T*_2_ and *D* were fitted using the following relations in Dynamic Center:

2 $$f\left(t\right)={I}_{0}*{e}^{-\frac{t}{T2}},$$

3 $$f\left(g\right)={I}_{0}*{e}^{-{\gamma }^{2}*{g}^{2}*{\delta }^{2}*\left(\Delta -\frac{\delta }{3}\right)*D},$$

As shown in Figs. 3C and S6, for the extraction of muscle texture features in the fish microstructure, the average value (AVE) and SD of *T*_2_ and *D* in the imaging area were calculated to evaluate the proton mobility level and variation in depth. The moving average value and first derivative were calculated, and the number of zero points in the first derivative was obtained; this value was named the “EDGE” of *T*_2_ and *D* from the imaging of fish muscle. The spectra of 2D chemical shift imaging (CSI)^40^ were observed using the pulse sequences in Fig. S7. The parameters were as follows: spectral width of 14,098 Hz and acquisition time of 0.29 s. Spectral data were processed using SMOOSY software developed by our team, and the projections of F2 were used for analysis.

### Machine learning, BN inference and statistical analysis

PCA, machine learning, bnlearn and correlation analysis were performed on the R platform. Machine learning was performed with the RF method. The RF models were evaluated with fivefold cross-validation. The RF models were evaluated with fivefold cross-validation. After the division and reservation of the test data, the data for each class used for modeling were randomly duplicated to reach the maximum sample number for classes 1 to 4 to eliminate bias. The generated data with equal sample numbers for each class were used as the modeling data. RF calculations were performed 5 times each with the test data for k1 to k5. The result files of RF (prediction accuracy and important variables identified by the Gini index) were generated, and the average values of RF1 to RF5 were calculated and used as the final RF results. BN inference was performed with the score-based hill-climbing learning algorithm implemented in the R package bnlearn. For the ecological category-dependent network, RF-learned metabolites with high Gini index were used as the input continuous variables. For the integrative network, RF-learned metabolites with high Gini index, the major soluble macromolecules, physical characteristics and 1D MRI-based phenotypic textural features were used as the input continuous variables, while the four ecological categories were used as input categorical variables. As the output data, all the probabilistic strengths between two variables were calculated. The connection with a probabilistic strength more than 0.5 was used in BN network construction. The importance of each variable was calculated as the sum of all the probabilistic strengths of each variable connected to the corresponding parent and child nodes. Data discretization was performed with Hartemink’s information-preserving discretization algorithm in the infotheo R package. The network was visualized using Gephi software (https://gephi.org/). Metabolic pathway enrichment analysis was performed using the free-web software MetaboAnalyst 4.0 (www.metaboanalyst.ca).

## Supplementary Information

Supplementary Table.

Supplementary Figures.

## Data Availability

The data that support the findings of this study are available from the corresponding author upon reasonable request.
