# Supplementary material for: Fish ecotyping based on machine learning and inferred network analysis of chemical and physical properties
Source: Sci Rep. 2021 Feb 12;11:3766. doi: 10.1038/s41598-021-83194-0 (PMC7881121; doi:10.1038/s41598-021-83194-0)
Supplement: Supplementary file 2 — Supplementary Figures. [file 41598_2021_83194_MOESM2_ESM.pdf]

Supplementary Materials

Fish ecotyping based on machine learning and inferred network analysis of chemical and physical properties

Feifei Wei[1], Kengo Ito[1], Kenji Sakata[1], Taiga Asakura[1], Yasuhiro Date[1], and Jun Kikuchi[*,1,2,3]

[1]RIKEN Center for Sustainable Resource Science, 1-7-22 Suehiro-cho, Tsurumi-ku, Yokohama 235-0045, Japan

[2]Graduate School of Medical Life Science, Yokohama City University, 1-7-29 Suehirocho, Tsurumi-ku, Yokohama 230-0045, Japan

[3]Graduate School of Bioagricultural Sciences and School of Agricultural Sciences, Nagoya University, 1 Furo-cho, Chikusa-ku, Nagoya 464-8601, Japan

[*]E-mail: jun.kikuchi@riken.jp

Table S1. Sample list of fish used in the present study (Attached excel file)

Unsupervised NMR-based metabolic profiling of fish muscle. Unsupervised principal component analysis (PCA) revealed that PC1 accounted for 14.08% of the variation in the NMR spectral data of the small molecular water-soluble metabolites of all fish muscle samples **(Fig. S1*A*)**. The positive direction of PC1 was dominated by the families of Gobiidae, Cyprinidae, Synodontidae, Aulopidae, and Engraulidae. Most of the fish species belonging to these families are freshwater (Cyprinidae) and brackish water (Gobiidae and Synodontidae) fishes. In contrast, the negative direction of PC1 was mainly dominated by marine fishes. Thus, the metabolic profile of the water-soluble components of fish muscle is mostly affected by the adaptation to salinity but not temperature, latitude or other factors related to water habitats. The loading plot showed that in the positive direction of PC1, the characteristic metabolites of freshwater and brackish fish muscles were mainly determined by the contents of taurine; the metabolites involved in the ornithine-urea cycle (OUC), such as *N*-acetyl-glutamate (NAG), urea, glutamate (Glu), glutamine (Gln), and arginine (Arg); and fish essential amino acids (EAAs), including valine (Val), leucine (Leu), isoleucine (Ile), and phenylalanine (Phe). In the negative direction of PC1, the characteristic metabolites of marine fish muscles were mainly associated with the contents of metabolites related to energy metabolism, such as creatine, lactate, the amine oxides trimethylamine-*N*-oxide (TMAO); the corresponding degradation products trimethylamine (TMA) and dimethylamine (DMA); and the ATP degradation products adenosine monophosphate (AMP), inosine monophosphate (IMP), and inosine **(Fig. S1*B*)**. These data from the view of fish muscle metabolism suggested that the freshwater and brackish water fishes had relatively strong ammonia detoxification ability, while marine fishes might have relatively strong athletic ability to meet the relevant ecological energy demands and tolerate the hydrostatic pressure conditions in the ocean. Notably, the metabolic diversity of 826 yellowfin gobies (*Acanthogobius flavimanus*) is shown on the PC2 axis, and this result explained 10.36% of the variance **(Fig. S1*C*)**. In accordance with our previous research on the temporal and spatial changes in a large sample size of yellowfin gobies, the loading plot of PC2 demonstrated that the characteristic water-soluble metabolites of yellowfin goby muscle were

mainly associated with the growth stage **(Fig. S1*D*)**. Intriguingly, PC3, which explained 4.82% of the variance, represented the depth distribution characteristics of the fishes **(Fig. S1*A*)**. The positive direction of PC3 was almost dominated by deep-living marine fishes in the mesopelagic zone (depth range 200-1000 m), such as Gadidae (*Theragra chalcogramma*, depth > 1280 m; *Gadus macrocephalus*, depth range 10-1280 m), Etmopteridae (*Etmopterus pusillus*, depth range 0-1070 m), and Macrouridae (*Coelorinchus japonicus*, depth range 300-1000 m). In contrast, the negative direction of PC3 was mainly dominated by shallow-living marine fishes in the epipelagic zone (depth < 200 m), such as Coryphaenidae (*Coryphaena hippurus*, depth range 0-85 m) and Scombridae (*Scomber japonicus*, depth range 0-300 m; *Thunnus orientalis*, depth range 1-550 m; *Sarda orientalis*, depth range 1-167 m; *Scomber australasicus*, depth range 87-200 m; *Auxis thazard*, depth < 50 m). The loading plot of PC3 illustrated the high contributions of TMAO and taurine, known as the osmolytes of marine fish, in the negative direction of PC3, and the high contributions of lactate and glucose were shown in the muscle of shallow-living fishes **(Fig. S1*B*)**. These results agreed with previous reports that the content of osmolytes, such as TMAO, increased with depth in bony fishes (teleosts) and was the primary factor that determines the living depth of fishes. A structural study revealed that TMAO might act as a nanocrowding particle agent to induce the stabilization of proteins and peptides by entropic stabilization mechanisms. Collectively, the nonbiased PCA model based on water-soluble muscle metabolites in a large set of fish samples indicated that the most important factors that affect the fish metabolism profile are environmental salinity (PC1), the growth stage (PC2) and habitat depth (PC3).

Figures

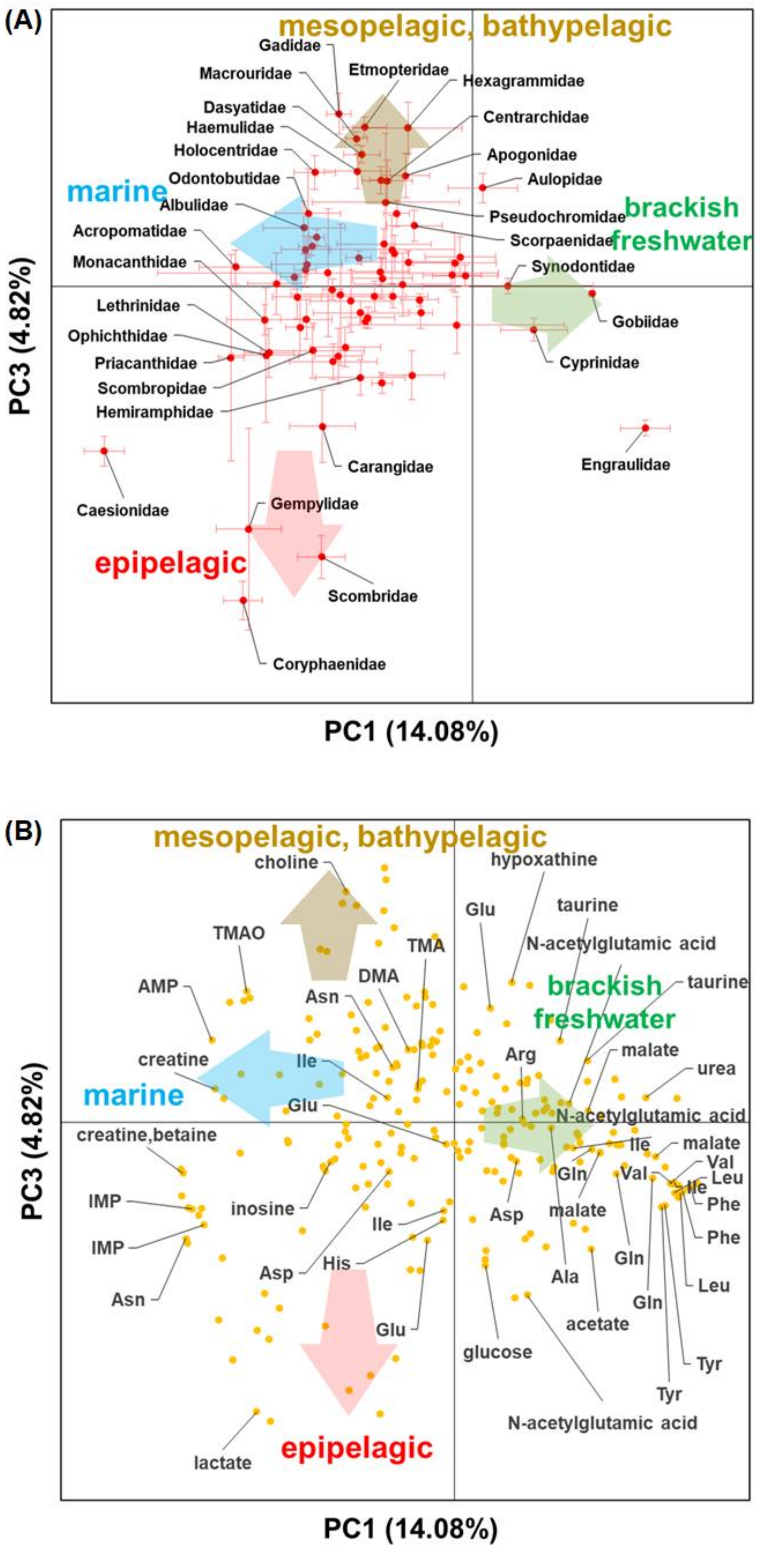

(to be continued)

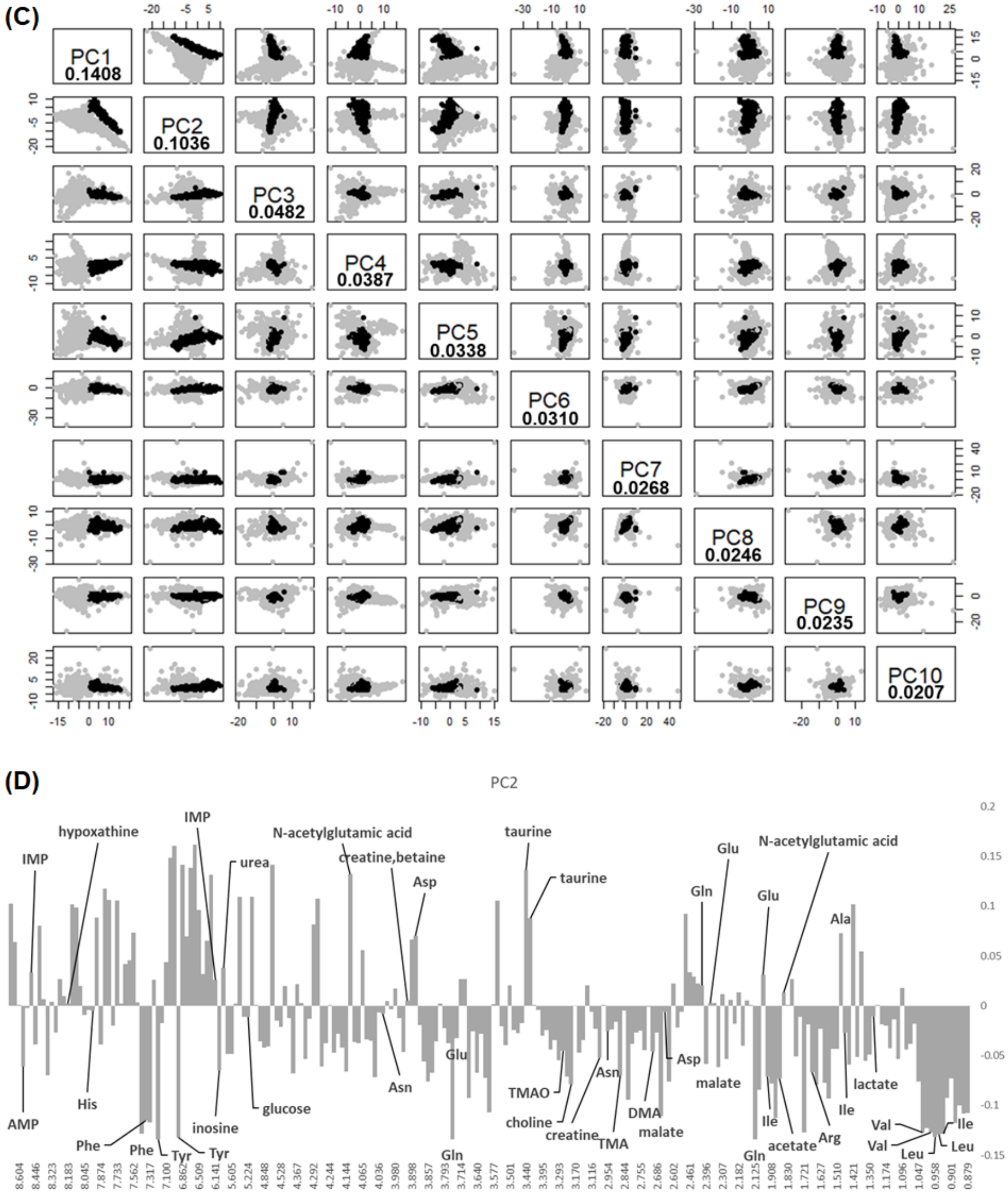

Fig. S1. NMR-based chemical profiling of fish muscle. **(A)** Score plot and **(B)** loading plot of PCA (PC1 vs. PC3) based on NMR spectral data for all fish muscle samples. **(C)** PCA score plots from PC1 to PC10 applied to the NMR-based metabolic profiles of fish muscles. The contribution degrees of PC1 to PC10 were 0.1408, 0.1036, 0.0482, 0.0387, 0.0338, 0.0310, 0.0268, 0.0246, 0.0235 and 0.0207, respectively. Plots in black indicate fish samples of yellowfin goby (*Acanthogobius flavimanus*); plots in gray indicate other fish samples in the preset study. **(D)** PCA loading plot of PC2. Signal assignment was performed using 1D and 2D NMR spectra based on our previous studies.

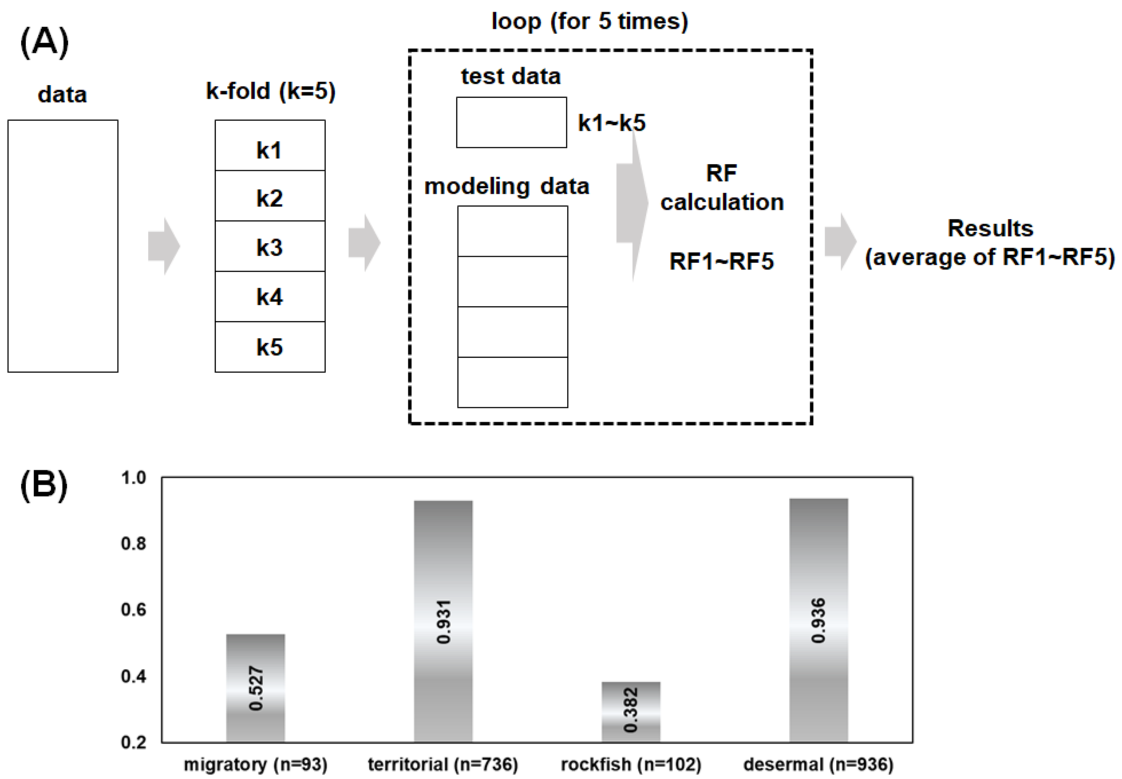

Fig. S2. (A) Conceptual diagram of the random forest (RF) used in the present study. The peak-picked and scaled matrix data from raw spectra were divided into 5 parts, one of which was used as the test data set, and the rest were used as modeling data. The RF calculations were performed 5 times by sequentially using k1 to k5 as the test data. The RF result files (prediction accuracy and important variables identified by the Gini index) were generated, and the average values of RF1 to RF5 were calculated and used as the final RF results. **(B)** The prediction accuracy of four ecological categories (migratory: n = 93; territorial, n = 736; rockfish: n = 102; demersal: n = 936) using RF without bias adjustment.

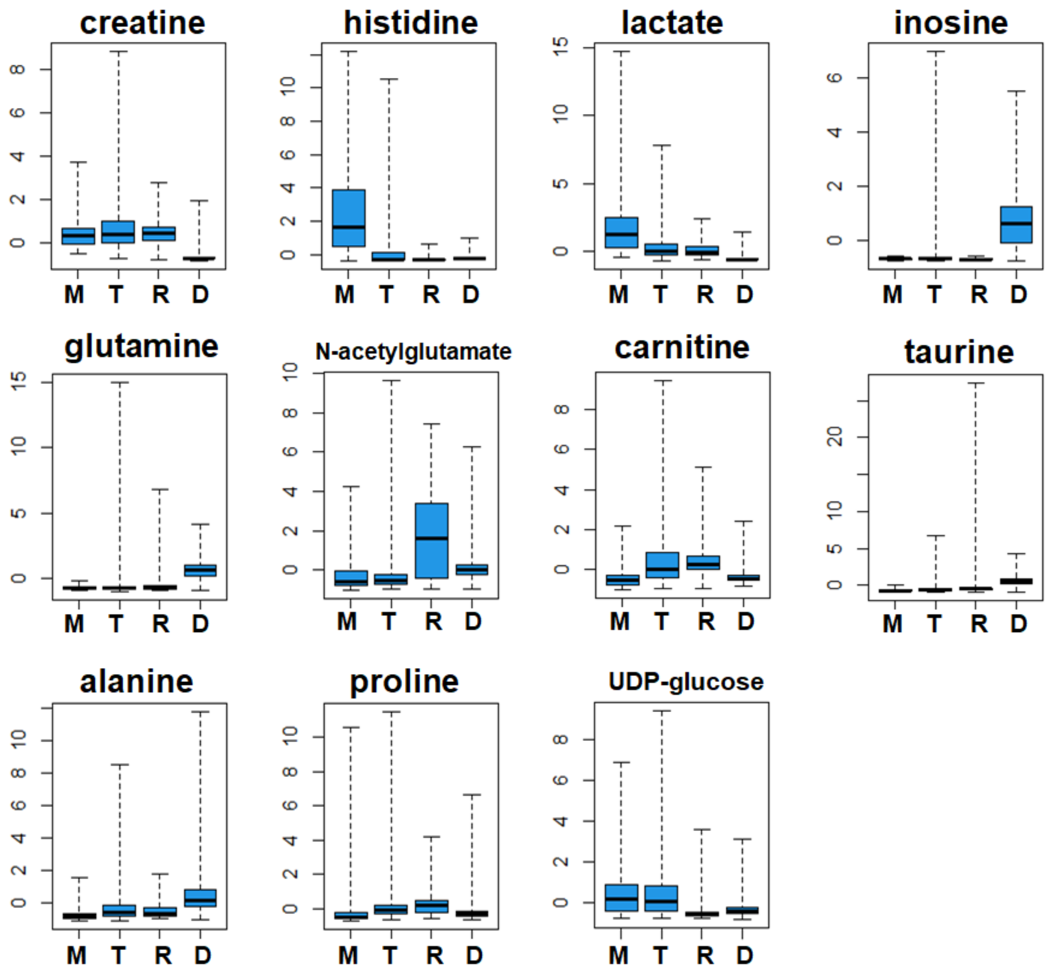

Fig. S3. The box plots for the highlighted muscle metabolites with a high Gini index in the RF prediction of the mobility patterns of fish. “M”, “T”, “R” and “D” indicate the fish mobility patterns of migratory, territorial, rockfish and demersal fish, respectively.

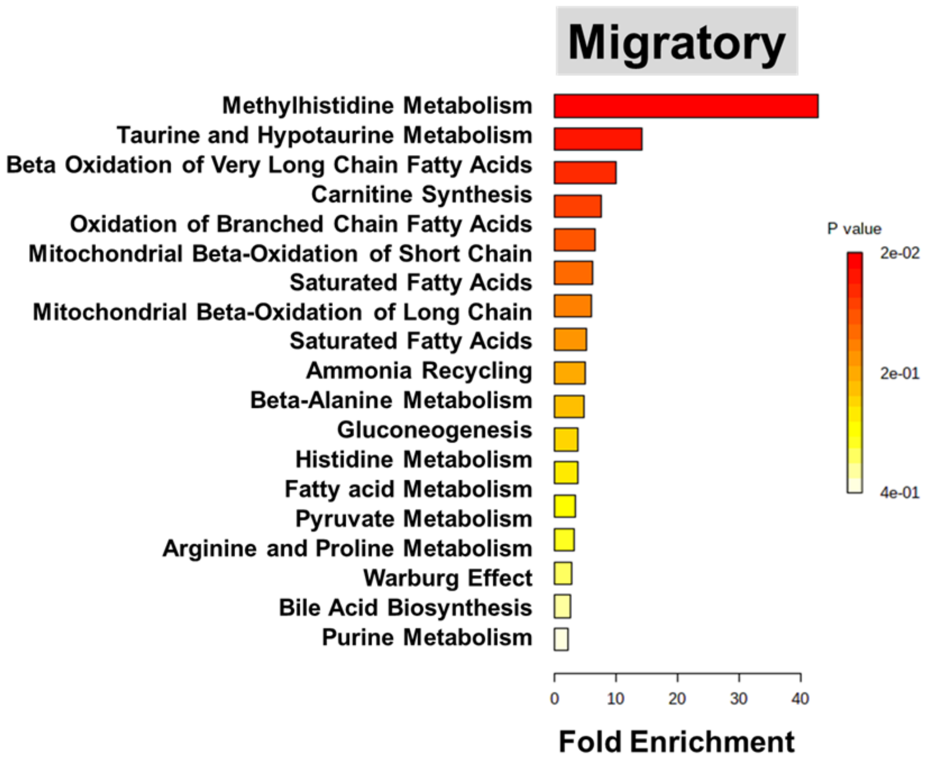

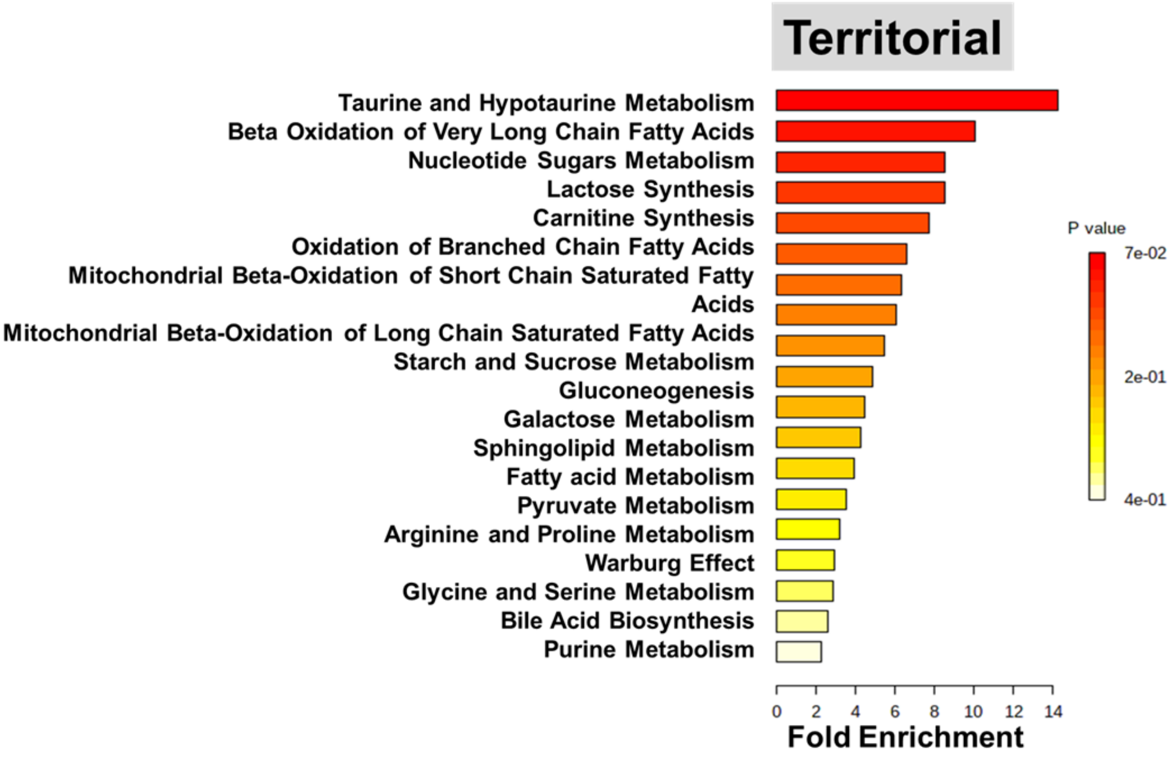

(to be continued)

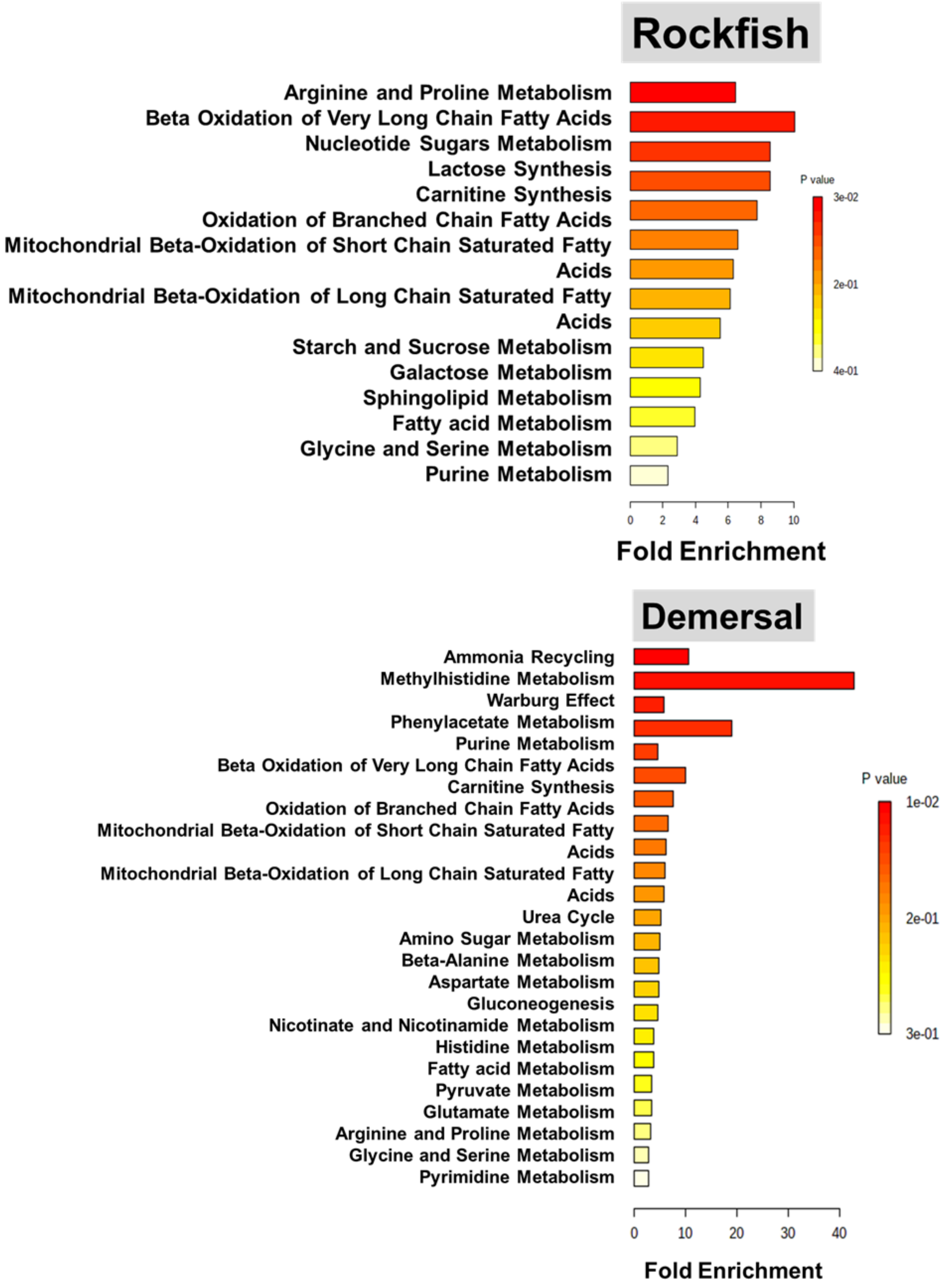

Fig. S4. KEGG metabolic pathway enrichment analysis of the top-6 metabolites ranked by the sum of probabilistic strength of the four ecological categories (migratory, territorial, rockfish, and demersal) using Metaboloanalyst.

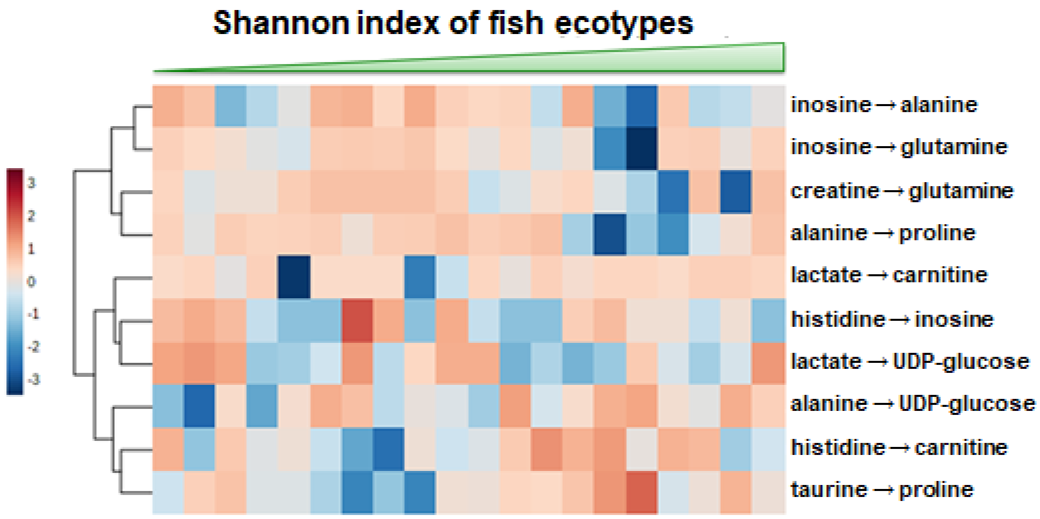

Fig. S5. Hierarchical clustering of the strength of the highly correlated metabolic dependence relations with the Shannon diversity index.

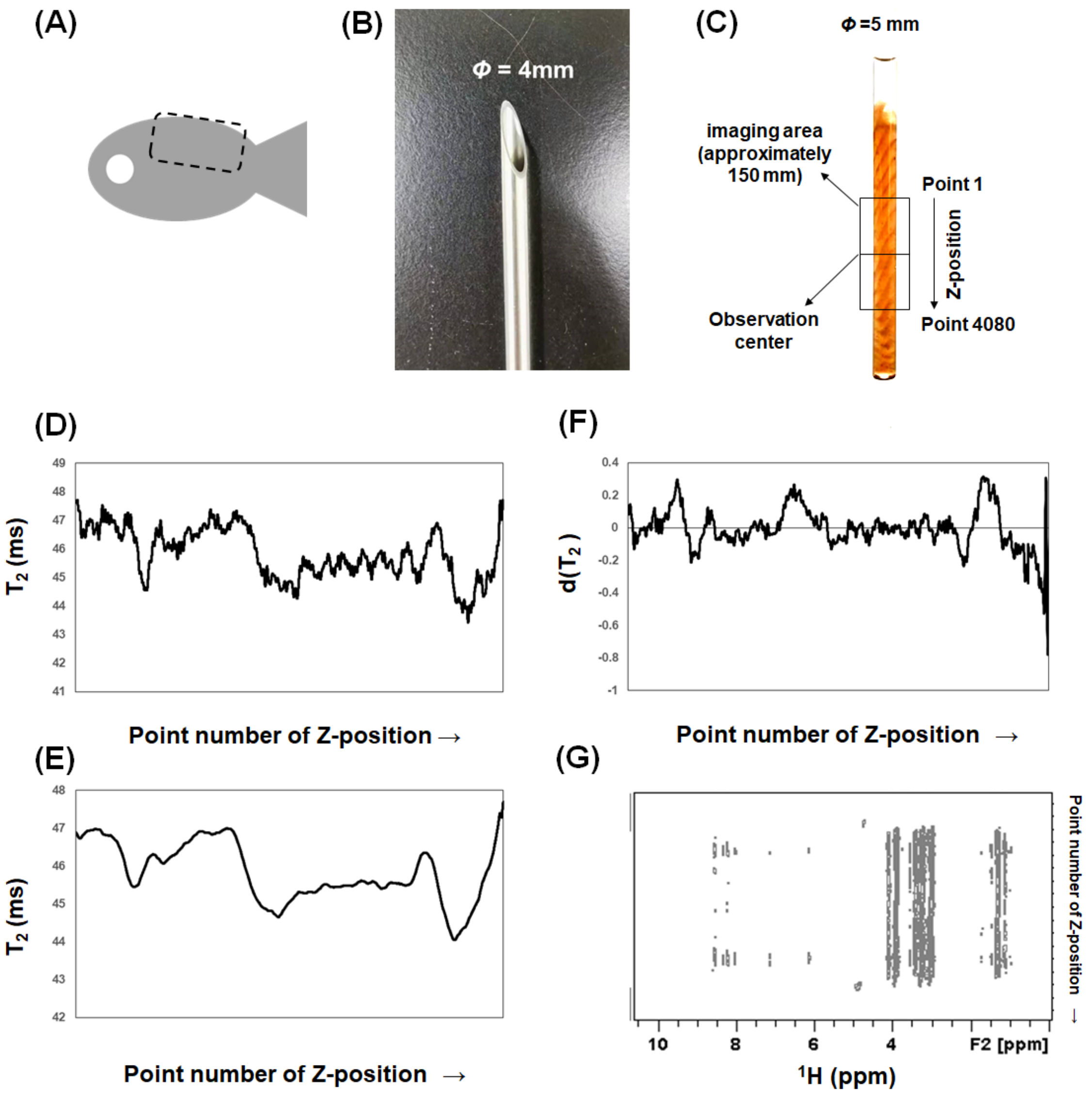

Fig. S6. (A) Fish muscle area (above the anal fin) used for 1D MRI-based intact observations. **(B)** A stainless pipe (Φ_{inside} = 4 mm) was used as a cutter, and **(C)** the drilled cylindrical muscle was inserted into a 5-mm NMR tube filled with KPi buffer. The observation area of the *Z*-axis gradient was approximately 150 mm of the equipped probe used in the present study. 1D MRI signals of proton density, **(D)** T_2 and diffusion coefficients were observed. After calculating the **(E)** moving averages of ±100 data points, **(F)** the first derivative of the curve was calculated to evaluate the "EDGE" (number of zero points in the first derivative) of the 1D imaging data. **(G)** Chemical shift imaging (CSI) of cylindrical fish muscle.

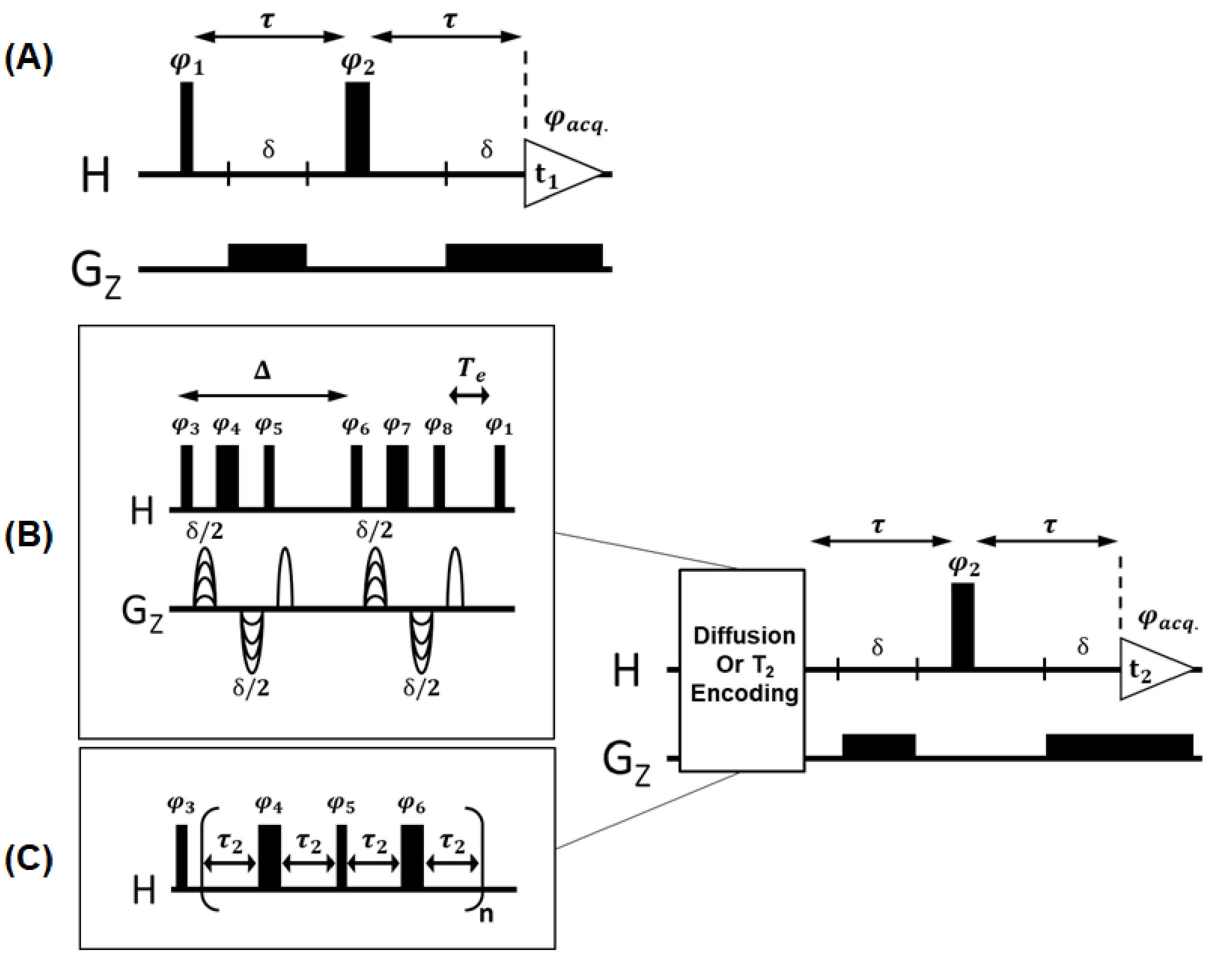

CYCLOPS

	**(A)** 1D	**(B)** DOSY	**(C)** PROJECT
φ_1	$(x)_4,(y)_4,(-x)_4,(-y)_4$	$(x)_4,(-x)_4,(y)_4,(-y)_4$	-
φ_2	$x,-x,(y,-y)_2,-x,x,y,-y,(-x,x)_2,-y,y$	$(x,-x)_2,(-x,x)_2,(y,-y)_2,(-y,y)_2$	x
φ_3	-	x	$x,-x$
φ_4	-	x	$(y)_2,(-y)_2$
φ_5	-	$(x)_2,(-x)_2$	$(y)_4,(-y)_4$
φ_6	-	$(x)_4,(-x)_4,(y)_4,(-y)_4$	$(y)_2,(-y)_2$
φ_7	-	x	-
φ_8	-	$(x,-x)_2,(-x,x)_2,(y,-y)_2,(-y,y)_2$	-
$\varphi_{acq.}$	$(x)_2,(-x)_2,(y)_2,(-y)_2$	$x,-x,(-x,x)_2,x,-x,-y,y,(y,-y)_2,-y,y$	$(x,-x)_2$

Fig. S7. Pulse sequences used in 1D MRI measurements of fish muscle. **(A)** 1D proton density; **(B)** diffusion coefficients; **(C)** T_2.
